# ENL reads histone β-hydroxybutyrylation to modulate gene transcription

**DOI:** 10.1093/nar/gkae504

**Published:** 2024-06-17

**Authors:** Chen Chen, Cong Chen, Aiyuan Wang, Zixin Jiang, Fei Zhao, Yanan Li, Yue Han, Ziping Niu, Shanshan Tian, Xue Bai, Kai Zhang, Guijin Zhai

**Affiliations:** The Province and Ministry Co-sponsored Collaborative Innovation Center for Medical Epigenetics, Key Laboratory of Immune Microenvironment and Disease (Ministry of Education), Department of Biochemistry and Molecular Biology, Tianjin Medical University, Tianjin 300070, China; The Province and Ministry Co-sponsored Collaborative Innovation Center for Medical Epigenetics, Key Laboratory of Immune Microenvironment and Disease (Ministry of Education), Department of Biochemistry and Molecular Biology, Tianjin Medical University, Tianjin 300070, China; The Province and Ministry Co-sponsored Collaborative Innovation Center for Medical Epigenetics, Key Laboratory of Immune Microenvironment and Disease (Ministry of Education), Department of Biochemistry and Molecular Biology, Tianjin Medical University, Tianjin 300070, China; The Province and Ministry Co-sponsored Collaborative Innovation Center for Medical Epigenetics, Key Laboratory of Immune Microenvironment and Disease (Ministry of Education), Department of Biochemistry and Molecular Biology, Tianjin Medical University, Tianjin 300070, China; The Province and Ministry Co-sponsored Collaborative Innovation Center for Medical Epigenetics, Key Laboratory of Immune Microenvironment and Disease (Ministry of Education), Department of Biochemistry and Molecular Biology, Tianjin Medical University, Tianjin 300070, China; The Province and Ministry Co-sponsored Collaborative Innovation Center for Medical Epigenetics, Key Laboratory of Immune Microenvironment and Disease (Ministry of Education), Department of Biochemistry and Molecular Biology, Tianjin Medical University, Tianjin 300070, China; The Province and Ministry Co-sponsored Collaborative Innovation Center for Medical Epigenetics, Key Laboratory of Immune Microenvironment and Disease (Ministry of Education), Department of Biochemistry and Molecular Biology, Tianjin Medical University, Tianjin 300070, China; The Province and Ministry Co-sponsored Collaborative Innovation Center for Medical Epigenetics, Key Laboratory of Immune Microenvironment and Disease (Ministry of Education), Department of Biochemistry and Molecular Biology, Tianjin Medical University, Tianjin 300070, China; The Province and Ministry Co-sponsored Collaborative Innovation Center for Medical Epigenetics, Key Laboratory of Immune Microenvironment and Disease (Ministry of Education), Department of Biochemistry and Molecular Biology, Tianjin Medical University, Tianjin 300070, China; The Province and Ministry Co-sponsored Collaborative Innovation Center for Medical Epigenetics, Key Laboratory of Immune Microenvironment and Disease (Ministry of Education), Department of Biochemistry and Molecular Biology, Tianjin Medical University, Tianjin 300070, China; The Province and Ministry Co-sponsored Collaborative Innovation Center for Medical Epigenetics, Key Laboratory of Immune Microenvironment and Disease (Ministry of Education), Department of Biochemistry and Molecular Biology, Tianjin Medical University, Tianjin 300070, China; Tianjin Key Laboratory of Digestive Diseases, Department of Gastroenterology and Hepatology, Medical University General Hospital, Tianjin Medical University, Tianjin 300070, China; The Province and Ministry Co-sponsored Collaborative Innovation Center for Medical Epigenetics, Key Laboratory of Immune Microenvironment and Disease (Ministry of Education), Department of Biochemistry and Molecular Biology, Tianjin Medical University, Tianjin 300070, China

## Abstract

Histone modifications are typically recognized by chromatin-binding protein modules (referred to as ‘readers’) to mediate fundamental processes such as transcription. Lysine β-hydroxybutyrylation (Kbhb) is a new type of histone mark that couples metabolism to gene expression. However, the readers that prefer histone Kbhb remain elusive. This knowledge gap should be filled in order to reveal the molecular mechanism of this epigenetic regulation. Herein, we developed a chemical proteomic approach, relying upon multivalent photoaffinity probes to capture binders of the mark, and identified ENL as a novel target of H3K9bhb. Biochemical studies and CUT&Tag analysis further suggested that ENL favorably binds to H3K9bhb, and co-localizes with it on promoter regions to modulate gene expression. Notably, disrupting the interaction between H3K9bhb and ENL via structure-based mutation led to the suppressed expression of genes such *MYC* that drive cell proliferation. Together, our work offered a chemoproteomics approach and identified ENL as a novel histone β-hydroxybutyrylation effector that regulates gene transcription, providing new insight into the regulation mechanism and function of histone Kbhb.

## Graphical Abstract

**Graphical Abstract:**
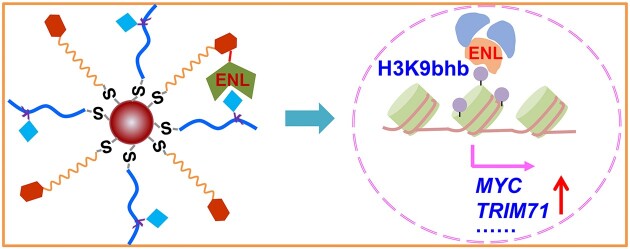

## Introduction

Histone post-translational modifications (hPTMs, e.g. lysine acetylation and methylation) are a group of fundamental epigenetic marks found in mammalian chromatin (1,2). With the development of mass spectrometry-based proteomics, a range of novel hPTMs has been reported, including various types of lysine acylation, such as crotonylation (Kcr) (3), succinylation (Ksucc) (4), 2-hydroxyisobutyrylation (Khib) (5), β-hydroxybutyrylation (Kbhb) (6) and lactylation (Kla) (7). Emerging evidence suggests that these hPTMs possess important roles in regulating cell processes, including gene transcription, DNA damage repair, replication and chromatin remodeling (8–11). Moreover, the hPTMs are typically considered as docking sites to execute these functions by recruiting various specific binding partners, referred to as readers (12–14). Following the first discovery of the bromodomain (BrD) as a reading module of acetyllysine (15), a number of readers such as YEATS, DPF and ZZ domains have been characterized for type- and site-specific readout of histone acetylation (16–20). However, downstream effectors capable of interpreting histone non-acetyl acylations such as β-hydroxybutyrylation are poorly understood.

Histone β-hydroxybutyrylation is a novel epigenetic regulatory mark that links metabolism to gene transcription. It is remarkably induced by β-hydroxybutyrate that is derived from starvation or diabetic ketosis of cells where cellular metabolism typically switches and the new steady-state balance exists (21). The mark may lead to particular transcription events that govern cell growth and development through associating with a set of up-regulated genes that distinguish histone acetylation, corroborating that histone Kbhb has distinct roles from histone Kac (6). Indeed, Zhang and co-workers (22) recently reported that BDH1-mediated histone Kbhb up-regulated stemness-associated genes, promoting propagation of hepatocellular carcinoma (HCC) cells. Similarly, the Huang group (23) uncovered that ketogenesis-generated histone Kbhb enhances gene expression and CD8^+^ T-cell memory development, coupling epigenetic modification with energy metabolism. In addition, Zheng and colleagues (24) showed that histone Kbhb regulates ferroptosis-suppressor gene expression in pancreatic acinar cells. However, how histone Kbhb regulates transcription of these gene remains largely elusive. Given the unique structure of Kbhb, traditional histone readers may lack sufficient ability to recognize this hPTM. It is one of the main reasons why readers of Kbhb have been unclear up to now. Thus, identification of readers that specifically recognize histone Kbhb is vital to understand the molecular mechanism of this epigenetic regulation.

Identification of hPTM effector proteins is challenging owing to the fact that the hPTM-mediated protein–protein interactions (PPIs) are weak and transient (25,26). Therefore, an affinity purification approach based on a mimic peptide (27) may not be applicable for the mining of hPTM readers, whereas photoaffinity probes can be an alternative method (28,29). We recently developed a self-assembled multivalent photoaffinity probe that we used for efficient and selective enrichment of histone Kcr readers and Khib erasers (30,31). In this study, we further design the probe and combine it with a quantitative proteomics approach to selectively profile binders of histone Kbhb. By using this method, the interactomes of β-hydroxybutyrylation of histone H3 lysine 9 (H3K9bhb) were determined, and ENL was identified as a novel H3K9bhb reader. Biochemical and molecular recognition studies and CUT&Tag analysis demonstrate that ENL recognizes H3K9bhb, and co-localizes with this mark on gene promoters. Disrupting the association between ENL and histone Kbhb by structure-guided mutation of Y78A in the YEATS domain reduced the recruitments of ENL to H3K9bhb-enriched peaks, resulting in the down-regulation of genes such as *MYC* that are responsible for tumorigenesis.

## Materials and methods

### Materials and instruments

Histone peptides with and without lysine trimetylation or β-hydroxybutyrylation modification were synthesized and purified by high-performance liquid chromatography (HPLC) by Beijing SciLight Biotechnology Co Ltd. Thiolated polyethylene glycol (HS-PEG) modified with benzophenone (HS-PEG-Bpa) was obtained from ToYong Biotech Ltd. Gold nanoparticles were purchased from BBI solutions. HPLC solvents used for mass spectrometric analysis of proteins were purchased from Thermo Fisher Scientific Ltd. Antibodies against ENL were obtained from Abcam. Matrix-assisted laser desorption ionization–time of flight mass spectrometry (MALDI-TOF MS) spectra were acquired by an Autoflex III TOF/TOF mass spectrometer (Bruker), and protein samples were identified by a Nano-LC-Q-Exactive Plus mass spectrometer (Thermo Fisher Scientific, Waltham, MA, USA).

### Synthesis and characterization of hPTM probes

For the fabrication of the probes, 2 mM peptides with or without PTMs and 1 mM HS-PEG-Bpa were mixed and added to the solution of gold nanoparticles (200 μl), and the mixture was shaken at room temperature for 12 h. Then, the functionalized gold nanoparticles were washed with H_2_O (50 μl) twice and the fabricated probes were stored at 4°C for further use. MALDI-TOF-MS and transmission electron microscopy (TEM) were used to confirm the probes.

### Extraction of nuclear proteins

Nuclear proteins were extracted from cells using nuclear extraction reagents (Nucleoprotein Extraction Kit, Sangon Biotech). Briefly, HepG2 cells were washed twice with phosphate-buffered saline (PBS; 0.1 M phosphate, 0.15 M NaCl, pH 7.2) and harvested into a tube (1.5 ml). After removing PBS, hypotonic buffer (protease inhibitor added beforehand) was added and incubated for 10 min on ice. Then, the solution was centrifuged at 800 *g* for 5 min and the supernatant was discarded. The precipitate was washed with hypotonic buffer in a mixer for 30 s followed by centrifugation at 2500 *g* for another 5 min. After centrifugation, the supernatant was removed. Next, the precipitate was resuspended in lysis buffer and incubated for 20 min. Finally, nucleoprotein was collected by centrifugation at 20 000 *g*for 10 min.

### Enrichment of endogenous binders by hPTM probes

The functionalized probes H3K9bhb and H3K9 were each incubated with 0.5 mg of nuclear extracts for 12 h at 4°C in a rotation wheel. Then, the mixture was irradiated at 365 nm on ice for 20 min using UVP Crosslinker (Analytik Jena). After centrifugation, the precipitate was washed twice with wash buffer 1 (4 M urea in PBS), followed by washing once with buffer 2 (50 mM Tris–HCl, pH 7.8, 200 mM NaCl, 2.5 mM KCl, 2.5 mM MgCl_2_, 1 mM ZnCl_2_) and once with wash buffer 3 (100 mM NH_4_HCO_3_). Next, the pellet was suspended in 50 μl of NH_4_HCO_3_ (100 mM), and trypsin (1 μg) was added to digest it overnight at 37°C. Finally, the mixture was centrifuged for 2 min and the supernatant was collected. Then, 5 mM dithiothreitol (DTT) was added and incubated with the obtained solution for 1 h at 65°C, followed by reaction with 15 mM iodoacetamide for 45 min and 30 mM cysteine for 30 min at room temperature. The resulting peptides were desalted with C18 material and further analyzed by HPLC-MS/MS.

### HPLC-MS/MS analysis

Each tryptic digest was redissolved in 7 μl of HPLC buffer A [0.1% (v/v) formic acid in water]. After centrifugation at 12 000 *g* for 2 min, the supernatant (5 μl) was injected into a Nano-LC system (EASY-nLC 1200, Thermo Fisher Scientific). Each sample was separated by a C18 column (50 μm inner diameter × 15 cm, 2 μm C18) with a 60 min HPLC gradient at a flow rate of 300 nl/min. The HPLC eluate was electrosprayed directly into an Orbitrap Q-Exactive Plus mass spectrometer (Thermo Fisher Scientific). The source was operated at 2.2 kV. The mass spectrometric analysis was carried out in a data-dependent mode with an automatic switch between a full MS scan and an MS/MS scan in the orbitrap. For full MS survey scan, the automatic gain control (AGC) target was 1e6, and the scan range was from 350 to 1750 with a resolution of 70 000. The 10 most intense peaks with charge state 2 and above were selected for fragmentation by higher energy collision dissociation (HCD) with a normalized collision energy of 27%. The MS2 spectra were acquired with 17 500 resolution. The exclusion duration for the data-dependent scan was 10 s, and the exclusion window was set at 1.6 Da.

### Data processing

The resulting MS/MS data were searched using Proteome Discoverer software (v2.1) with an overall false discovery rate (FDR) for peptides of <1%. Proteins demonstrating an average score (*n* = 3) of <4 were removed from the identification list. Peptide sequences were searched using trypsin specificity and allowing a maximum of two missed cleavages. Carbamidomethylation on cysteine was specified as a fixed modification. Oxidation of methionine and acetylation on the protein N-terminus were set as variable modifications. Mass tolerances for precursor ions were set at ±10 ppm for precursor ions and ±0.02 Da for MS/MS.

### Western blot analysis

The enriched proteins were loaded and separated on an 8% sodium dodecylsulfate–polyacrylamide electrophoresis (SDS–PAGE) gel and transferred to a nitrocellulose membrane (Pall Corporation, 0.22 μm). The membranes were first blocked with 5% non-fat milk and incubated with primary antibodies (ENL) overnight at 4°C at a dilution of 1:500. After washing, the membranes were incubated with goat anti-rabbit horseradish peroxidase (HRP)-conjugated secondary antibody (1:5000) for 2 h at room temperature.

### AutoDock analysis

AutoDock 4.0 was used to dock the peptides H3K9bhb and H3K9ac into the structures of ENL. The structure of ENL was taken from the Protein Data Bank (PDB) (PDB: 5J9S). Before the docking simulation, the peptide was placed into the middle of the ENL surface as the start point of docking. The parameters for docking were set as follows: the Lamarckian genetic algorithm (LGA) runs were set at 100, and the maximum number of energy evaluations was set at 25 million. The simulation box was fixed at the center of the substrate and the box size was set at 80 Å in all three dimensions. The conformation with the highest binding energy of small molecules was considered as the best conformation.

### Recombinant protein expression and purification

Recombinant ENL_YEATS_ (residues 1–148) was cloned into the ET28b-SUMO vector and expressed with an N-terminal 6× His-SUMO tag in *Escherichia coli* strain BL21(DE3) (ZOMANBIO) and induced overnight by 0.5 mM isopropyl-β-d-thiogalactoside at 16°C in LB medium. Overnight-induced cells were collected and suspended in lysis buffer: 50 mM Tris–HCl, pH 8.0, 500 mM NaCl and 1 mM phenylmethylsulfonyl fluoride (PMSF). Then the cells were lysed using an ultrasonic crusher. After centrifugation, the supernatant was applied to a nickel column, and proteins were eluted with 300 mM imidazole. The resultant proteins were treated with ULP enzyme overnight for His-SUMO tag removal. The tag-free ENL_YEATS_ proteins were further concentrated to ∼8 mg/ml, and stored at −80°C. ENL_YEATS_ mutants were generated using QuikChange (Genstar) methods and verified by sequencing. Recombinant mutant ENL_YEATS_ proteins were expressed and purified with essentially the same method as for wild-type (WT) ENL_YEATS_.

### Isothermal titration calorimetry (ITC)

The ITC experiments were carried out on the MicroCal PEAQ ITC instrument (Malvern Instrument) at 15°C. The protein at 100 μM was dropped into the 1000 μM peptide segment for 19 consecutive drops, and the resulting titration curve was drawn using the ‘one set of binding sites’ model and the Origin 7.0 program. The protein concentration was determined by UV absorption at 280 nm. The peptide concentration was measured by nanodrop.

### Analytical gel filtration

Analytical gel filtration experiments for the detection of protein–peptide interactions were carried out using an analytical Superdex S200 (Cytiva) gel filtration column with a flow rate of 0.5 ml/min on an Äkta purifier system (GE Healthcare) in a 20 mM HEPES pH 7.5, 150 mM NaCl buffer at 4°C. The protein and peptide H3K9bhb were incubated at a ratio of 1:1.5 at 4°C and the total applied sample volumes were 300 μl in both cases. Protein elution was followed by recording the UV adsorption at 280 nm.

### Fluorescence microscopy

After transfecting with WT-ENL or its mutants, and growth for 48 h, cells were washed in PBS in triplicate followed by being fixed for 15 min in PBS containing 4% (w/v) paraformaldehyde. After washing, the HepG2 cells were then permeabilized for 30 min with PBS containing 0.25% Triton X-100, blocked with 3% bovine serum albumin (BSA) in PBS for 2 h in room temperature, and incubated with mouse anti-DDDDK-Tag monoclonal antibody (Abclonal, AE005) and H3K9bhb (PTMBio, PTM-1250RM) antibody overnight at 4°C. After washing with PBS, cells were incubated with secondary antibodies coupled to AlexaFluor 488 or 647 for 1.5 h in room temperature, and then stained with 4′,6-diamidino-2-phenylindole (DAPI) for 5 min. Finally, cells were imaged using a Zeiss LSM 800 microscope with a ×63 oil objective. Images were merged using Adobe Photoshop and analyzed by ImageJ.

### CUT&Tag

CUT&Tag is operated according to the manufacturer's instructions for the Hyperactive Universal CUT&Tag Assay Kit for Illumina (TD903, Vazyme Biotech). In brief, HepG2 cells expressing WT-ENL or Mutant-ENL were collected and counted for incubation with pre-treated concanavalin A (ConA) beads. Subsequently, cells were resuspended in an antibody buffer and incubated overnight at 4°C with the corresponding primary antibody of Flag or anti-H3K9bhb. The secondary antibodies were diluted in appropriate proportions and incubated with the cells at room temperature. Subsequently, pA/G-Tnp transposons were rotated and incubated with the samples for 1 h to activate the translocase fragment DNA and extract the DNA. The DNA library was constructed with the TruePreP Index Kit V2 for Illumina (TD202, Vazyme Biotech). The library was purified by VAHTS DNA Clean Beads (N411, Vazyme Biotech) and sequenced by Illumina novaseq 150PE.

### ChIP-qPCR analysis

Chromatin precipitation (ChIP) analysis was performed essentially as described below. Briefly, HepG2 cells were cross-linked with 1% formaldehyde for 10 min and stopped with 125 mM glycine. The isolated nuclei were resuspended in nuclei lysis buffer and sonicated using a Bioruptor Sonicator (Diagenode). The samples were immunoprecipitated with 2–4 μg of the appropriate antibodies overnight at 4°C. Protein A/G beads were added and incubated for 1 h, and the immunoprecipitates were washed twice each with low-salt, high-salt and LiCl buffers. Eluted DNA was reverse-cross-linked, purified using a polymerase chain reaction (PCR) purification kit (Genestar) and analyzed by quantitative real-time PCR on the ABI 7500-FAST System using the Power SYBR Green PCR Master Mix (Genestar). Statistical differences were calculated using a two-way unpaired Student's *t*-test. The primers used for qPCR are listed in Supplementary Table S3.

### RNA-seq analysis

RNA-seq samples were sequenced using the Illumina Hiseq 2500, and raw reads were mapped to the human reference genome (hg19) and transcriptome using the RNA-seq unified mapper. Read counts for each transcript were calculated using HTseq v0.6.1 employing default parameters 54. Differential gene expression analyses were performed using the ‘exactTest’ function in edgeR v3.055. Gene Ontology (GO) analysis was performed using the DAVID Bioinformatics Resource 6.756. The volcano plot was drawn by using the ggplot2 package (https://cran.r-project.org/package=ggplot2) in the R computing environment.

### Cell Counting Kit-8 (CCK-8) assay

Cell Counting Kit-8 (CCK-8) was used to measure cell proliferation. HepG2 cells were seeded into 96-well plates at a density of 3000 cells per well. Every 24 h for 7 days, 10 μl of the CCK-8 solution was added to each well with incubation for 2 h at 37°C. The absorbance of each well was then measured at 450 nm using a microplate reader (Thermo Fisher Scientific). Finally, the numbers of living cells over 5 days were plotted in a graph to calculate the cell proliferation rate.

### Cell migration assay

The migration assays were performed using a transwell chamber. After transfection for 24 h, HepG2 cells were seeded in transwell chambers at 7 × 10^4^ cells per well. Forty hours later, the cells which had migrated through the filter were fixed and stained with 2% crystal violet. For each chamber, the mean of the migrated cell number from five randomly fields was calculated and displayed in the plot.

## Results

### Development of a quantitative chemical proteomics approach for mapping hPTM binders

**Figure 1.**
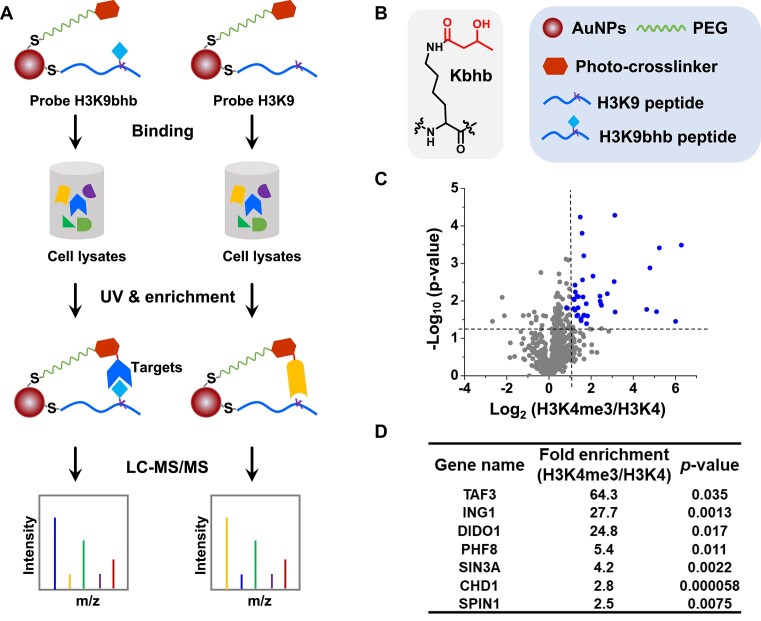
Proteomic profiling of hPTM interactors using photoaffinity probes. (**A**) Schematic workflow for enrichment and identification of hPTM target proteins by comparative proteomics along with self-assembly multivalent photoaffinity probes. (**B**) Structure of lysine β-hydroxybutyrylation. AuNPs, gold nanoparticles; PEG, polyethylene glycol. (**C**) Volcano plot of protein enrichment ratio and statistical analysis from proteomic experiments (*n* = 3) by using probes H3K4me3 and H3K4. Blue hits indicate that proteins meet the criteria (ratio ≥ 2.0 and *P*-value ≤ 0.05). (**D**) Several known H3K4me3 reader proteins identified with high confidence.

In order to selectively and robustly identify hPTM interactors, we envisaged a chemoproteomics strategy that coupled a multivalent photoaffinity probe with quantitative proteomics. We designed probe H3K9bhb that contains Kbhb-installed peptide (mimic histone Kbhb) and photo-cross-linker-linked PEG, based on the spacer of the cross-linker and the mark, leading to improvement in the efficiency of target identification. Furthermore, the multivalent effect of the photo-cross-linker produces multiple labeling events contributing to capture of weak PPIs. Based on the above, we prepared probe H3K9bhb through a self-assembled technique while a control probe H3K9, containing lysine 9 instead of Kbhb, was also synthesized to minimize non-specific binders and improve the determination of H3K9bhb interatomes (Figure 1A, B).

To test whether the strategy could be used to identify hPTM readers, we first set up comparative proteomic experiments to capture target proteins of H3K4me3, a well-established histone mark associated with actively transcribed genes (32). Similar to H3K9bhb, probe H3K4me3 and probe H3K4 were, respectively, prepared and incubated with cell lysate, and subsequently subjected to UV irradiation by which cross-linking between the probe and its targets could occur. After removing non-specific binders by stringent washing, the captured proteins were enriched by centrifugation and digested by on-bead trypsinization, followed by detection with HPLC-MS/MS to quantify protein abundance. Finally, the spectral counts (33,34) detected for each protein were used to calculate the enrichment ratios for proteins identified by probes H3K4me3 and H3K4 (Figure 1A). We performed three biological replicates to reliably identify binders enriched by probe H3K4me3, and proteins with a maximal mean of < 2.0 spectral counts were discarded. For the remaining proteins, we used an averaged enrichment ratio (H3K4me3/H3K4) of 2.0 (*n* = 3) as the cut-off to exclude non-specific binding proteins. Furthermore, *a t*-test between probe H3K4me3 and H3K4 was conducted to generate a set of proteins meeting a *P*-value < 0.05 as high-confidence targets. The identified proteins were then displayed by volcano plots as (log_2_) of the enrichment ratio against their statistical significance [–log_10_(*P*-value)] (Figure 1C; Supplementary Table S1).

As a result, 43 protein hits that meet the above criteria were identified, and the GO analysis on these proteins demonstrated that prominent among them are those enriched in chromatin binding, transcription factor binding and transcription regulator activity (Supplementary Figure S1), demonstrating that these proteins may be read by H3K4me3 to mediate cell functions such as gene expression. As expected, our analysis of proteins identified by the probe found several known readers of H3K4me3, such as TAF3 (35), PHF8 (36) and CHD1 (37), displaying 2.5- to 64.3-fold preference for the probe H3K4me3 over probe H3K4 (Figure 1D). The result demonstrates that our approach is capable of identifying endogenous HPTM readers from cell lysates.

### Identification of ENL as an H3K9bhb binder through the chemoproteomic approach

**Figure 2.**
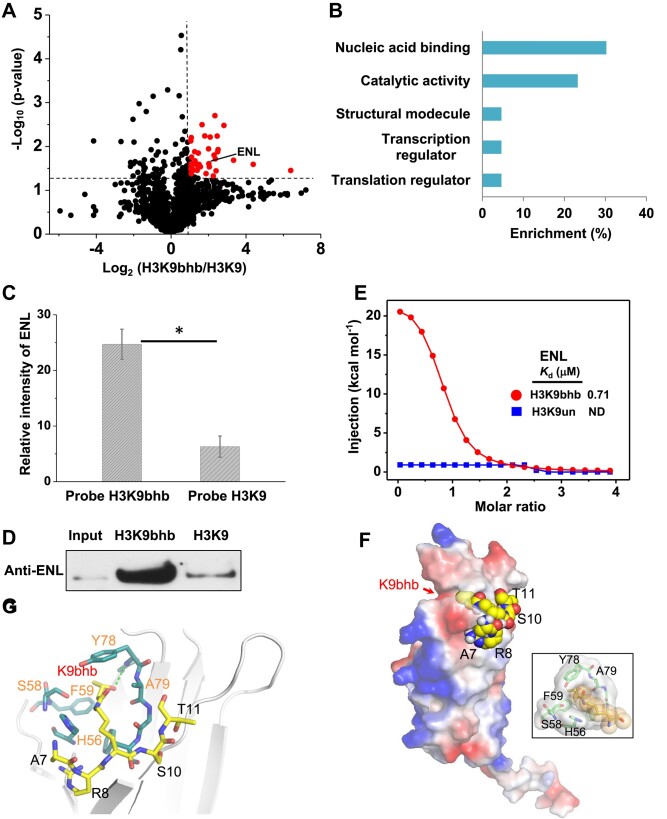
Identification of ENL as a H3K9hbb-binding protein using a chemoproteomics approach. (**A**) Volcano plot of protein fold enrichment and *P*-value from proteomic experiments (*n* = 3) by using probes H3K9hbb and H3K9. Red hits are proteins with a ratio ≥ 2.0 and *P*-value ≤ 0.05.(**B**) Molecular function analysis of H3K9bhb targets (red hits in A) by GO. (**C**) Parallel reaction monitoring (PRM) mass spectrometry and (**D**) western blotting analysis for the captured binders by probes H3K9hbb and H3K9. (**E**) ITC fitting curves of the ENL titrated with unmodified (H3K9un) or β-hydroxybutyryl histone H31-17 peptides (H3K9bhb). (**F** and **G**) Molecular recognition analysis of H3K9bhb read by ENL. Electrostatic potential surface view of the ENL space filled by H3K9bhb peptide. Bottom right, close-up view of the H3K9bhb-binding pocket of ENL (F). (**G**) The bonding network between H3K9bhb peptide and ENL. Hydrogen bonds are shown as green dashes. Key residues of ENL are depicted as blue sticks and labeled in orange; the H3K9bhb peptide is shown as yellow sticks and labeled in black.

We next applied this validated chemoproteomics strategy to identify targets of H3K9bhb, a histone mark associated with gene transcription (22). Recently, the enzymes that are responsible for removing or installing this modification have been discovered (38,39), while readers which prefer this mark remain largely unknown. Thus, it is of great importance to mine readers of H3K9bhb in order to decipher its biological effects. Herein, we developed probe H3K9bhb and H3K9, confirmed by MS and TEM data (Supplementary Figures S2 and S3), to uncover putative binders of the mark in cellular lysates. The probe H3K9bhb or H3K9 was first incubated with cell extracts, followed by UV-irradiation and stringent washing to remove non-specific binding proteins. Then, the enriched proteins were digested and subjected to LC-MS/MS analysis. Similarly, to exclude the false-positive binders, we also generated a volcano plot and screened proteins with a fold enrichment (probe H3K9bhb/H3K9) ≥2 and a *P*-value ≤ 0.05. As shown in Figure 2A and Supplementary Table S2, 43 proteins, identified from the pulldowns by probe H3K9bhb, satisfied the criteria. We next employed GO analysis on these potential binding proteins regarding their molecular function distributions, and found that major proteins are enriched with nucleic acid binding, catalytic activity and transcription regulator activity (Figure 2B).

Considering that nucleic acid binding was the top-ranked pathway and the related proteins may function through binding to histones, we continued to focus on the proteins involved in this cluster. After ruling out several RNA-binding proteins, we further concentrated on four targets (ZNF787, TFCP2, ENL and GTF2H4) that are associated with chromatin binding. Further domain analysis indicated that only ENL possesses the binding modules of acetylated histone (40); we therefore speculated that the H3K9bhb mark may be recognized by ENL, and selected it for further investigation.

### ENL efficiently binds to H3K9bhb through its YEATS domain *in vitro*

We next decided to investigate the putative interaction between ENL and the mark by using parallel reaction monitoring (PRM) MS, which has been reported as a targeted proteomic method for accurate quantitative measurement of proteins (41). Our result revealed that ENL exhibits a 3.9-fold enrichment for probe H3K9bhb compared with probe H3K9 (Figure 2C), which is in line with the proteomics profiling data. In addition, analytical gel filtration of ENL with or without peptide H3K9bhb showed two obvious separating peaks (Supplementary Figure S4), suggesting the binding of ENL and H3K9bhb. Next, to further confirm ENL–H3K9bhb interaction, we performed photoaffinity enrichment from a cell lysate followed by western blotting analysis of proteins isolated by probes H3K9bhb and H3K9, respectively. As expected, probe H3K9bhb captured ENL more efficiently than probe H3K9, validating that ENL could directly and selectively interact with H3K9bhb (Figure 2D).

In light of the ENL YEATS domain being a reader of histone acetylation (40), we proposed that ENL might recognize H3K9bhb via its YEATS domain. To test this hypothesis, we recombinantly expressed the YEATS domain of ENL (ENL_YEATS_), and performed an ITC assay using histone H3K14 (1–17) peptides, with binding dissociation constants (*K*_d_) of 0.71 μM, 8.44 μM and not detected (ND) for H3K9bhb, H3K9ac and unmodified peptides, respectively (Figure 2E; Supplementary Figure S5A). Notably, compared with H3K9ac, ENL_YEATS_ revealed an ∼12-fold increase in affinity for H3K9bhb. Furthermore, pulldown assays using biotinylated peptides showed that the YEATS domain of ENL exhibited a higher binding affinity toward H3K9bhb than H3K9ac (Supplementary Figure S5B). In addition, we also implemented ITC detection using H3K9bhb peptide with other YEATS domain-containing proteins (AF9, GAS41 and YEATS2), and measured a *K*_d_ of 11.0 μM, 6.7 μM and ND, respectively (Supplementary Figure S5C), which indicated that H3K9bhb may selectively recognize YEATS module proteins. Together, these results established that ENL favorably bound to H3K9bhb via its YEATS domain.

Moreover, to decipher the molecular basis for how ENL recognizes the H3K9bhb mark, we docked the binding of ENL_YEATS_ (PDB: 5J9S) with H3K9bhb peptide. As shown in Figure 2F and G, H3K9bhb inserts into the binding pocket formed with ENL_YEATS_ residues, such as H56, S58, F59 and Y78, in which an aromatic sandwich cage for K9bhb recognition is clamped by aromatic residues such as F59 and Y78. In addition to electrostatic and hydrophobic contacts, the recognition of H3K9bhb peptide by ENL is stabilized through a hydrogen bond of the Y78 residue and the hydroxyl group within β-hydroxybutyrylamide, differing from H3K9ac that is facilitated by a hydrogen bond between acetylamide and the Y78 main chain. This suggested that there may be different mechanisms for recognizing H3K9bhb and H3K9ac, thereby contributing to distinct affinities for ENL (Supplementary Figures S5D and S6). Collectively, these findings demonstrate that ENL is able to preferably recognize H3K9bhb relying on some key residues such as Y78, as a potential reader of this histone mark.

### ENL is intracellularly associated with H3K9bhb

**Figure 3.**
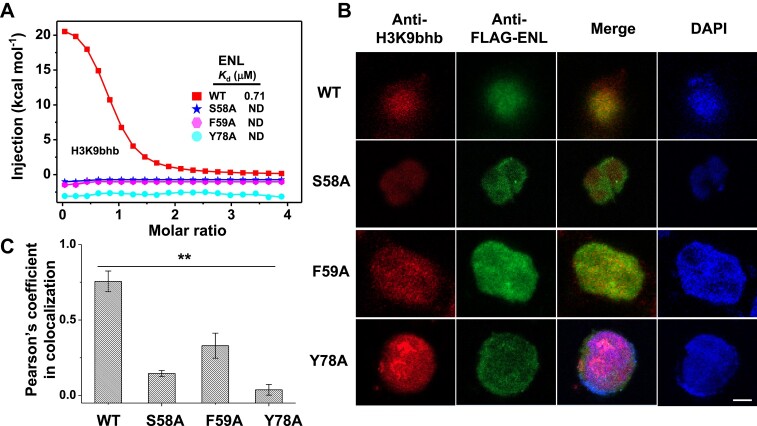
ENL mutagenesis and co-localization studies. (**A**) ITC titration fitting curves of ENL mutants with H3K9bhb peptide. (**B**) Representative IF images of cells treated with antibodies against FLAG (red) or H3K9bhb (green). FLAG-tagged ENL or its mutants were transfected into cells for IF analysis. (**C**) Quantification of the FLAG-ENL or its mutants co-localized with H3K9bhb using Pearson's correlation analysis.

To assess the association of ENL with H3K9bhb in cells, we set up ENL mutagenesis and immunofluorescence (IF) assay. Structure-guided mutagenesis was designed to alanine mutation of the Khbb-surrounding residues including S58, F59 and Y78. Subsequently, ITC assays revealed that the affinity of H3K9bhb binding for all mutations (S58A, F59A and Y78A) dramatically dropped in comparison with WT-ENL (Figure 3A). We then performed IF imaging determination to investigate their co-localization in cells transfected with WT-ENL or FLAG-tagged mutants, followed by reaction with antibodies against FLAG or H3K9bhb. As shown in Figure 3B and C, FLAG-tagged ENL and H3K9bhb rendered an even distribution pattern in the nucleus, and the signals resulting from them were merged with a high Pearson correlation coefficient of 0.76, thereby validating their co-localization in cells. By contrast, the value was significantly reduced in the ENL mutants, which indicates the disruption of the association owing to the mutation. Together, these observations suggest that ENL interacts with H3K9bhb via key residues of its YEATS domain in cells.

### ENL co-localizes with H3K9bhb across the whole genome

ENL is associated with acute myeloid leukemia, but its relationship with HCC remains unclear. Utilizing gene expression profiling interactive analysis (GEPIA), we found that ENL in HCC was remarkably up-regulated, and the high expression level of ENL was linked to short overall survival (Supplementary Figures S7 and S8). Based on this, we proposed that ENL may recognize H3K9bhb to modulate gene transcription, potentiating progression of HCC cells.

**Figure 4.**
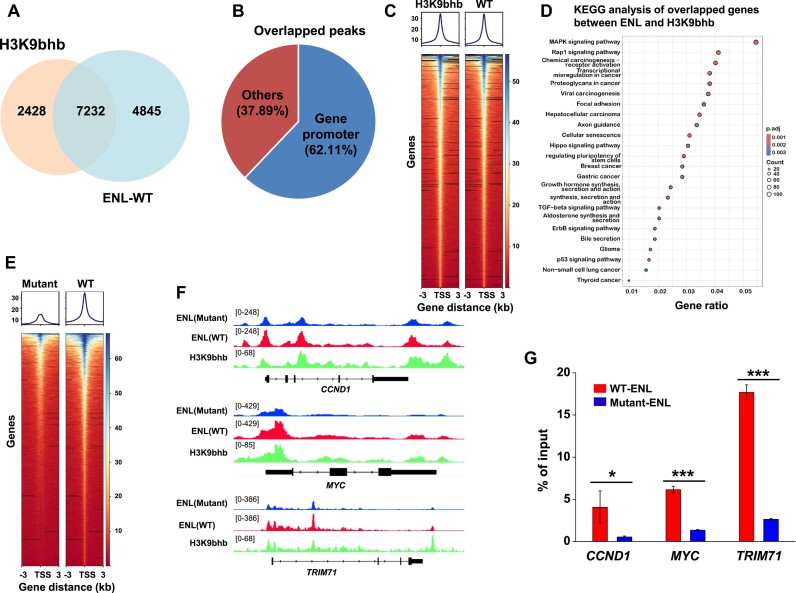
ENL co-localizes with H3K9bhb across the whole genome through CUT&Tag analysis. (**A**) Venn diagram showing the overlap of H3K9bhb- and ENL-occupied genes in cells. (**B**) The distribution of overlapping peaks within genes. (**C**) The heatmap illustrating that H3K9bhb globally co-localized with ENL at promoter regions. (**D**) The bubble graph showing the enrichment pathways in the KEGG analysis of the overlapping genes in ENL and H3K9bhb CUT&Tag results. (**E**) Averaged genome-wide occupancies of WT-ENL and Mutant-ENL (Y78A) around TSSs. (**F**) The genome browser view of WT-ENL, Mutant-ENL (Y78A) and H3K9bhb at the CCND1, MYC and TRIM71 locus, respectively. The *y*-axis represents CUT&Tag intensity. (**G**) The quantitative results by ChIP-qPCR assay for the selected genes. Data are shown as means (±SEM) of two independent experiments.

To investigate this, we firstly implemented CUT&Tag assays in HepG2 cells to test whether ENL links H3K9bhb in the native chromatin context. The cells stably expressing Flag-tagged ENL were immunoprecipitated using Flag antibodies or H3K9bhb antibodies, respectively. By high-throughput sequencing of these CUT&Tag experiments, we identified 9660 H3K9bhb-enriched peaks and 12 077 ENL-bound peaks, 7232 of which were co-occupied by both H3K9bhb and ENL (Figure 4A). Notably, the overlapping peaks were mainly positioned within gene promoter regions (62.1%) and others were mainly localized in the intergenic regions such as enhancers (Figure 4B), suggesting that H3K9bhb may associate with ENL to modulate gene transcription. Furthermore, the heatmap and average distribution of H3K9bhb and ENL in the promoter regions revealed a strong enrichment at transcription start sites (TSSs) and adjacent regions within 3 kb (Figure 4C). Genome browser views of the CUT&Tag signals of selected genes, which related to tumorigenesis, further confirmed the co-localization of ENL with H3K9bhb in TSSs (Figure 4F). Furthermore, Kyoto Encyclopedia of Genes and Genomes (KEGG) pathway analysis showed that ENL-bound and H3K9bhb-TSSs-marked genes were involved in the regulation of gene transcription, mitogen-acivated protein kinase (MAPK) signaling, focal adhesion, and so on (Figure 4D).

Given that ENL enables recognition of histone acetylation and crotonylation marks, we further explored whether there are genes co-occupied by H3K9ac-ENL, H3K9cr-ENL and H3K9bhb-ENL interactions through CUT&Tag detection. Consequently, we identified 1946 peaks marked by these modifications, among which the peaks were mainly localized at promoter regions and the genes resulting from these peaks were enriched in a variety of functional processes (Supplementary Figure S9). Importantly, there are a number of peaks that are specifically occupied by H3K9bhb-ENL, suggesting potential distinctive outputs mediated by this mark (Supplementary Figure S9A). Furthermore, H3K9ac rather than H3K9cr was marked at the H3K9bhb-ENL-enriched genes (such as *MYC*) (Supplementary Figure S9D), indicating the putative synergistic effect by histone acetylation on H3K9bhb-regulated gene transcription.

To further validate the dependence of ENL-binding peaks on the YEATS domain, we performed CUT&Tag assays with cells expressing Mutant-ENL (Y78A). As shown in Figure 4E, compared with WT-ENL, the average promoter region occupancies of Mutant-ENL were dramatically reduced, suggesting that ENL binds to these gene regions relying on the YEATS domain. Consistently, the Y78A mutation greatly disrupted ENL’s association with H3K9bhb at the selected gene promoter regions shown by ChIP-qPCR analysis (Figure 4F, G). Together, these findings demonstrate that ENL co-localizes with H3K9bhb across the genome (mainly on gene promoters), while the ENL_YEATS_ mutation attenuates the correlation.

### ENL interacts with H3K9bhb to regulate gene transcription depending on the YEATS domain

**Figure 5.**
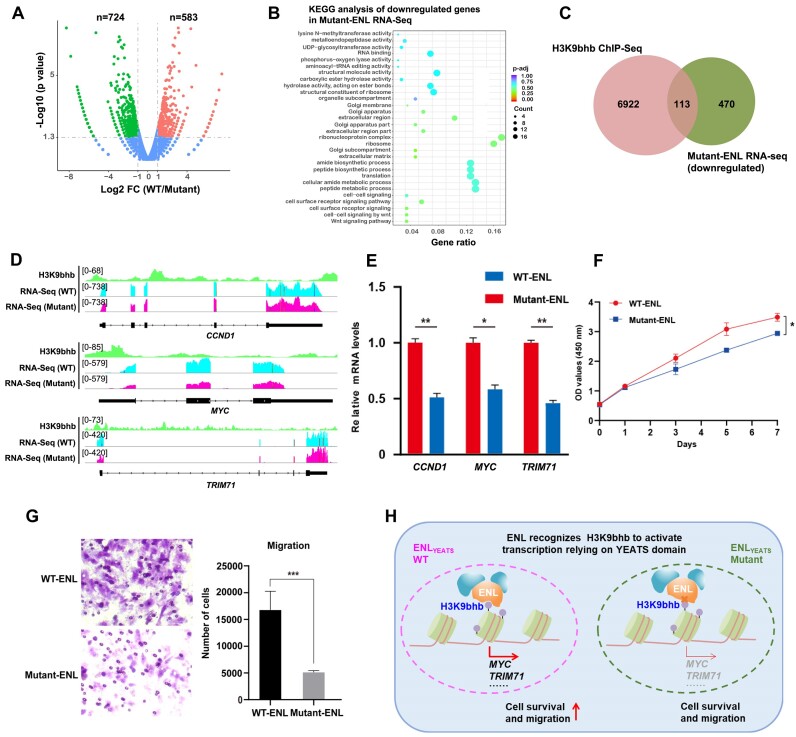
ENL correlates with H3K9bhb to regulate gene expression and cell survival relying on the YEATS domain. (**A**) Volcano plots of RNA-seq results of cells expressing WT-ENL compared with cells harboring Mutant-ENL. The up-regulated genes are colored in red, and down-regulated genes are shown in green. (**B**) The bubble graph showing the enrichment pathways in the KEGG analysis of the up-regulated genes in ENL RNA-seq compared with the ENL mutation group. (**C**) Venn diagram displaying the overlap of H3K9bhb-occupied and Mutant-ENL-down-regulated genes in cells. (**D**) The browser view of H3K9bhb CUT&Tag and ENL and its mutant RNA-seq track showing that mutation of ENL leads to reduced occupation at H3K9bhb-enriched TSSs, and down-regulates the expression of CCND1, MYC and TRIM71. (**E**) qRT-PCR analysis of the expression of the indicated genes in cells as in (D). (**F**) Cell proliferation assay in cells expressing WT-ENL or its mutants (Y78A). (**G**) Cell migration ability was determined by transwell assay in cells as in (F). **P < 0.01; ***P < 0.001. (**H**) The proposed mechanism whereby H3K9bhb recruits ENL to chromatin, and associates with it at gene promoters to boost transcription.

It was previously reported that ENL could activate oncogenic gene transcription through the link with histone acetylation. Given the association between ENL and H3K9bhb at gene promoters, we wondered if the ENL–H3K9bhb interaction could regulate gene expression. To this end, we performed RNA-seq by ectopically expressing WT-ENL or Mutant-ENL in HepG2 cells. Through the cut-off of log_2_ (fold change)>1 and *P*-value < 0.05, our result showed that the Mutant-ENL leads to suppressed expression of 583 genes (mainly included in the regulation of lysine writer activity, RNA binding, and so on) (Figure 5A, B), suggesting that ENL could be recruited to these genes to modulate transcription via the YEATS domain.

To exclude the potential effect of histone acetylation on the regulation of gene expression, we integrated H3K9bhb-occupied genes with those down-regulated by Mutant-ENL to define the overlapping genes as targets of H3K9bhb-ENL recognition. As a result, 113 genes satisfied this criteria, and responded for both H3K9bhb and ENL (Figure 5C). As expected, H3K9bhb and ENL CUT&Tag with RNA-seq track displayed that Mutant-ENL at H3K9bhb-enriched promoters results in down-regulation of the selected genes (Figure 5D). Notably, their transcript levels were verified by reverse transcription–quantitative PCR (RT–qPCR) analysis (Figure 5E). Furthermore, CCK-8 detection and transwell assay showed that mutation of ENL led to the remarkable reduction of cell proliferation and migration capacity (Figure 5F, G). Collectively, our results reveal a model where ENL reads H3K9bhb relying on its YEATS domain at gene promoters and recruits other factors to chromatin, to foster expression of genes such as *MYC* that are crucial for cell proliferation and tumorigenesis (Figure 5H).

## Discussion

In this study, we developed a quantitative chemoproteomics approach based on multivalent photoaffinity probes to capture hPTM-binding proteins. Using this strategy, we identified a number of known H3K4me3 readers, validating the effectiveness and advantage of the method. Moreover, we extended this strategy to mine the putative binders of H3K9bhb, a newly uncovered histone mark, and for the first time identify ENL as a novel target of this mark.

As a novel epigenetic mark, histone β-hydroxybutyrylation plays a key role in gene transcription. However, the involved regulation mechanism remains largely elusive. Given that histone readers serve as the core element of such epigenetic regulation, the identification of ENL provides a new opportunity to reveal the regulatory mechanism of histone Kbhb. Based on H3K9bhb chemoproteomics findings and biochemical and molecular docking studies, we revealed that ENL is capable of binding to H3K9bhb relying on its YEATS domain, in which the Y78 residue as the key site interacts with the β-hydroxybutyrate group to contribute to the recognition. Indeed, further CUT&Tag coupled with RNA-seq analysis revealed that the ENL-Y78A mutation disrupts the association with H3K9bhb on the promoters of genes such as *MYC* that are essential for tumorigenesis. Hence mutation of Y78A at ENL in clinical cancer samples may be explored as a promising candidate marker.

ENL has already been reported as a histone acetylation and crotonylation reader that modulates gene transcriptional programs. Here we also queried the candidate targets co-occupied by H3K9ac, H3K9cr, H3K9bhb and ENL, and demonstrated that there are numerous peaks exclusively marked by H3K9bhb-ENL in addition to the overlapping peaks, which indicated the potential distinctive gene transcription events regulated by H3K9bhb. Intriguingly, we also found that H3K9ac was enriched in the gene promoter regions of H3K9bhb-regulated genes such as *MYC*. Condisidering a higher binding affinity of H3K9bhb (*K*_d_ = 0.71 μM) than H3K9ac (*K*_d_ = 8.44 μM) towards ENL as well as lower peaks marked by H3K9bhb compared with H3K9ac, we surmise that histone Kbhb marks are likely to modulate transcription of these genes by competing for the binding of ENL with Kac, implying that H3K9bhb may function synergistically with H3K9ac through the recognition of ENL.

The work describes a mechanism of histone β-hydroxybutyrylation recruitment of ENL to gene promoters to activate transcription in cancer cells. Further investigations could assess whether the mechanism is available in other cells including starvation-induced liver cells. Future studies are also expected to determine the role of β-hydroxybutyrate in modulating histone Kbhb and thereby boosting cell proliferation.

In principle, we present a general chemoproteomic approach for capture of hPTM targets and identify ENL as a new reader of H3K9bhb. We envisage that our work will be useful for elucidating epigenetic regulatory mechanisms underlying histone marks in cells.

## Supplementary Material

gkae504_Supplemental_File

## Data Availability

The mass spectrometry proteomics data have been deposited to the ProteomeXchange Consortium via the PRIDE (42) partner repository with the dataset identifier PXD051689. RNA-sequencing and CUT&Tag data are available through GEO (GSE266268, 266269).
