# Supplementary material for: ENL reads histone β-hydroxybutyrylation to modulate gene transcription
Source: Nucleic Acids Res. 2024 Jun 17;52(17):10029–39. doi: 10.1093/nar/gkae504 (PMC11417371; doi:10.1093/nar/gkae504)
Supplement: gkae504_Supplemental_File [file gkae504_supplemental_file.pdf]

Supplementary Information for

ENL reads histone β-hydroxybutyrylation to modulate gene transcription

Chen Chen[1,†], Cong Chen[1,†], Aiyuan Wang[1,†], Zixin Jiang[1,†], Fei Zhao[1], Yanan Li[1], Yue Han[1], Ziping Niu[1], Shanshan Tian[1], Xue Bai[1], Kai Zhang[1,2,*], and Guijin Zhai[1,*]

[1] The Province and Ministry Co-sponsored Collaborative Innovation Center for Medical Epigenetics, Key Laboratory of Immune Microenvironment and Disease (Ministry of Education), Department of Biochemistry and Molecular Biology, Tianjin Medical University, Tianjin 300070, China

[2] Tianjin Key Laboratory of Digestive Diseases, Department of Gastroenterology and Hepatology, Medical University General Hospital, Tianjin Medical University, Tianjin 300070, China

* To whom correspondence should be addressed. Tel: +86 022 83336833; Email: zhaiguijin@tmu.edu.cn

Correspondence may also be addressed to Kai Zhang. Tel: +86 022 83336833; Email: kzhang@tmu.edu.cn

† The first, second, third and fourth authors should be regarded as Joint First Authors.

Supplementary Figures

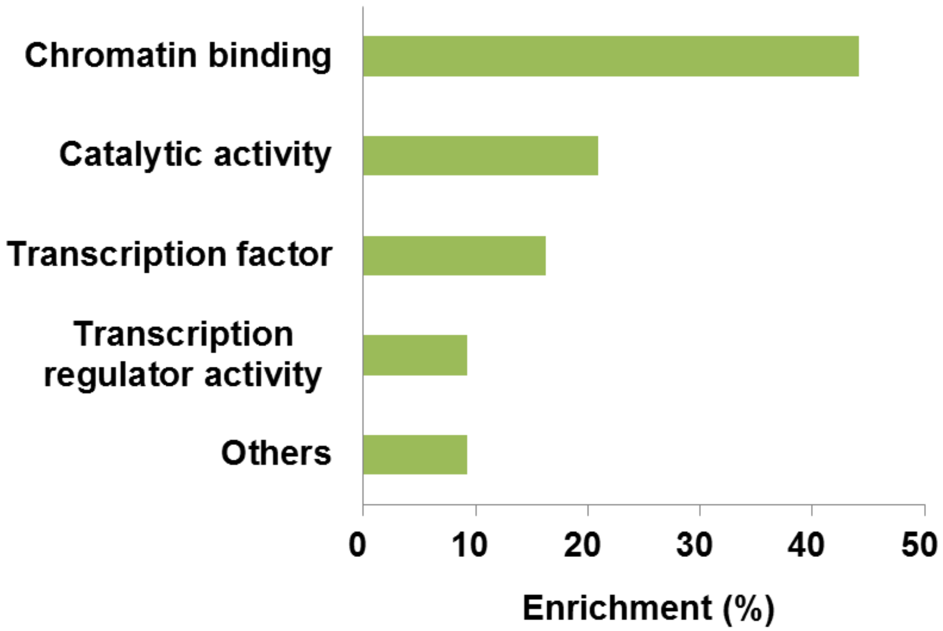

Figure S1. Molecular function analysis of H3K4me3-binding partners by gene ontology (GO).

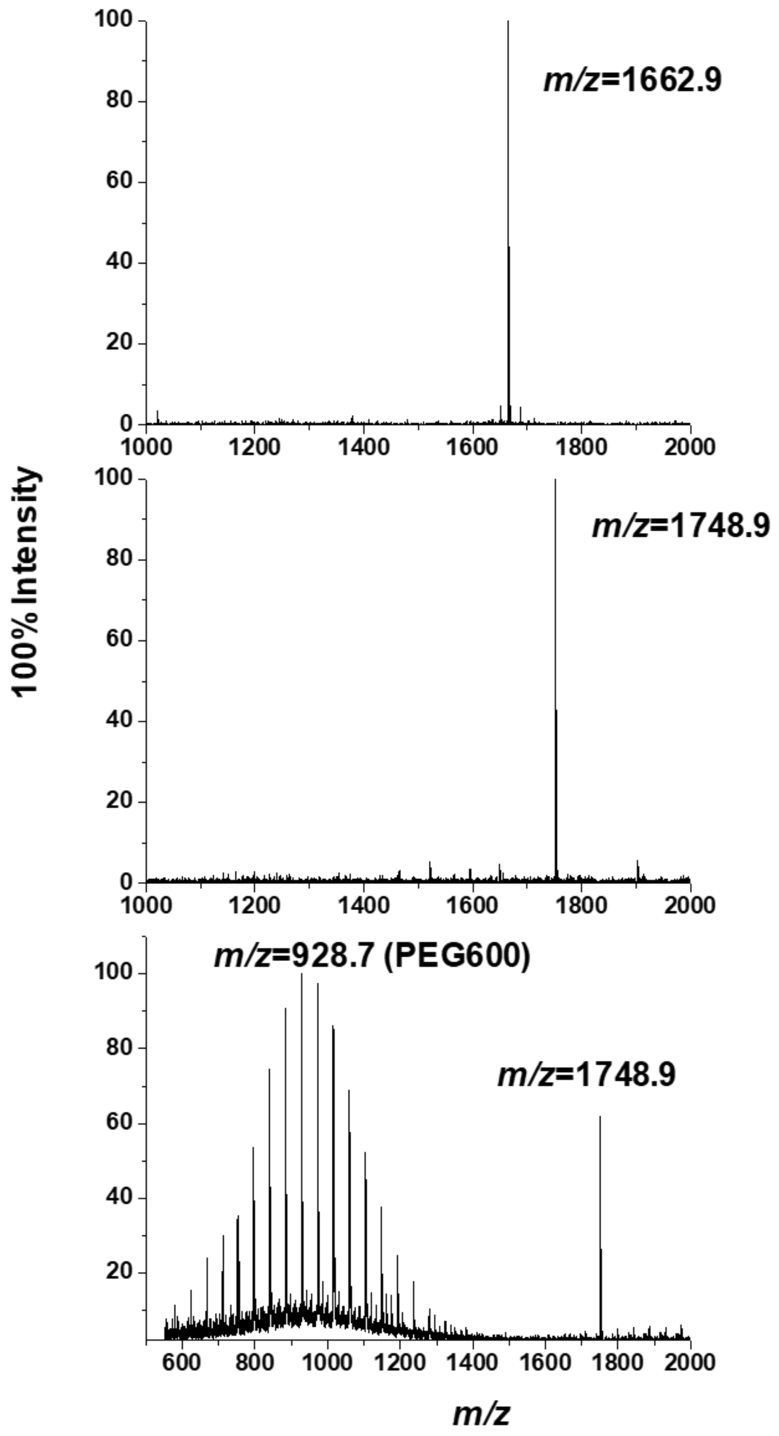

Figure S2. MALDI-TOF MS spectra of (A) histone peptide H3K9 and (B) H3K9bhb assembled to AuNPs and (C) probe H3K9bhb (with PEG600 conjugation). The above functionalized AuNPs was suspended with 3 μL matrix solution. The mixture was spotted onto the 384 sample plates for MALDI-TOF MS analysis.

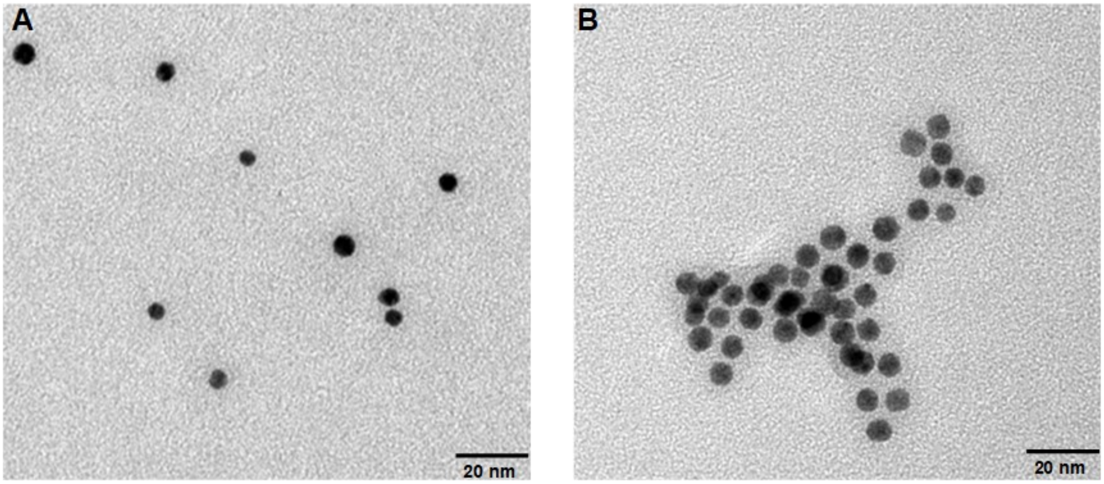

Figure S3. Representative TEM images of (A) bare AuNPs and (B) probe H3K9bhb. The images were obtained using Hitachi-HT7700 transmission electron microscope.

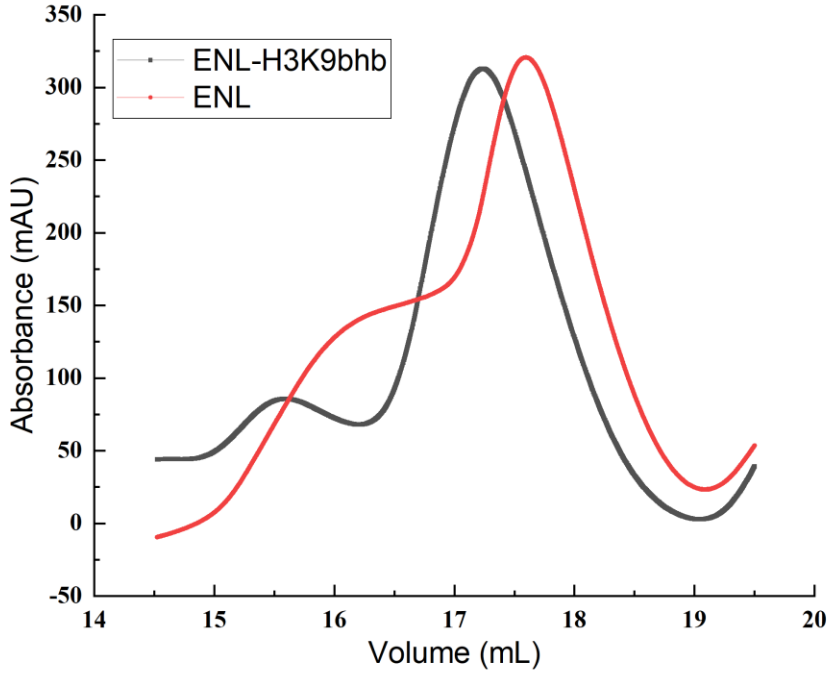

Figure S4. Analytical gel filtration of the ENL with and without H3K9bhb. ENL was incubated with or without peptide H3K9bhb at ratio of 1:1.5, and the elution was detected at 280 nm.

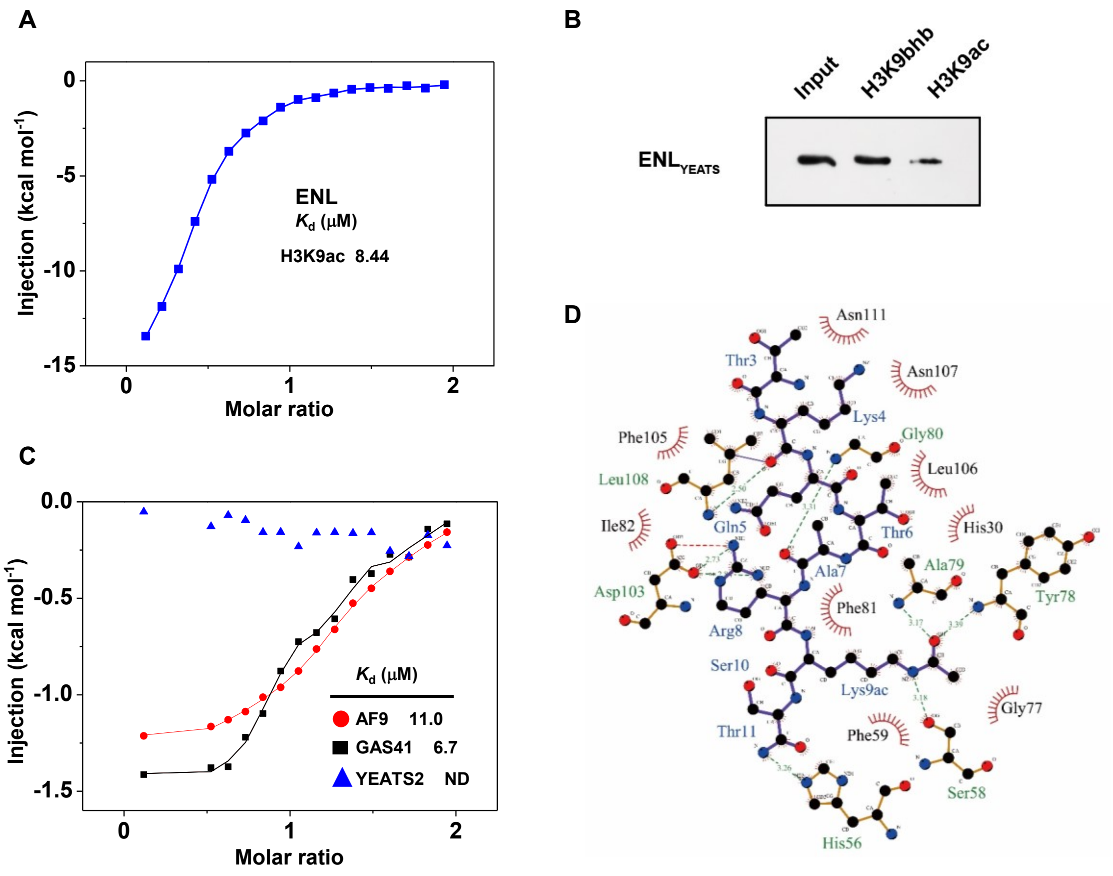

Figure S5. (A) ITC titration fitting curves of ENL mutants with H3K9bhb peptide. (B) Western blot analysis of histone peptide pulldowns with ENL$_{\text{YEATS}}$ and the indicated biotinylated peptides. Biotinylated H3K9bhb and H3K9ac were respectively incubated with ENL$_{\text{YEATS}}$ in binding buffer. Streptavidin beads were then added to the mixture and analyzed by SDS-PAGE and western blotting. (C) ITC titration fitting curves of the other YEATS domain-containing proteins (AF9, GAS41, and YEATS2) with H3K9bhb peptide. (D) LigPlot diagram showing critical contacts of H3K9ac within the binding pocket of ENLYEATS. Dashed lines, hydrogen bonds; Curved brushes, hydrophobic interactions.

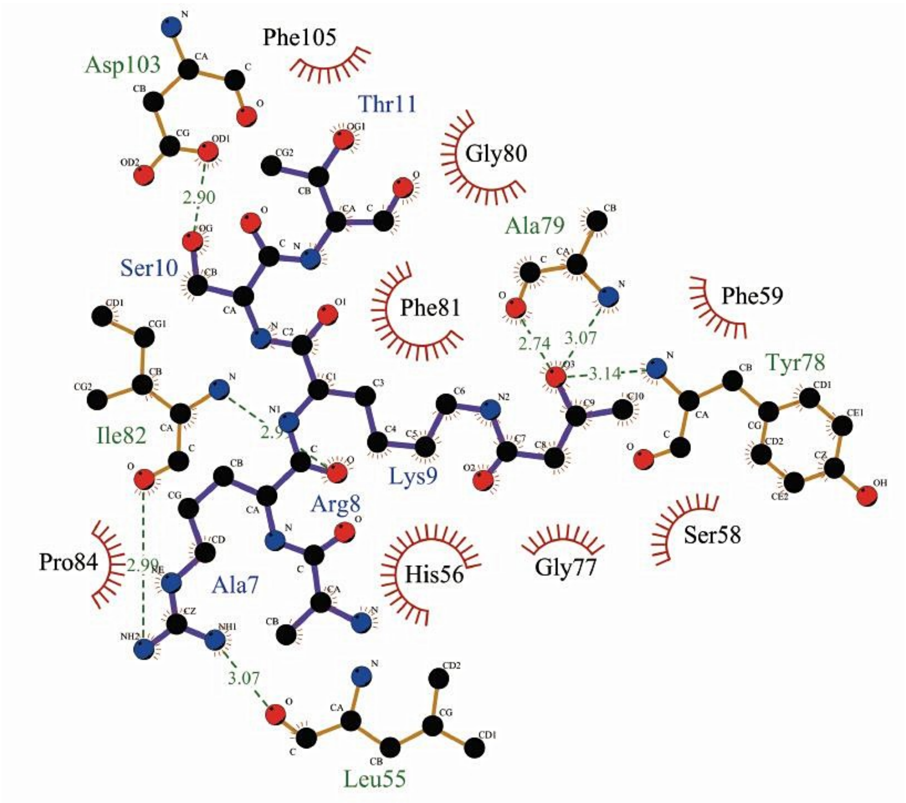

Figure S6. LigPlot diagram showing critical contacts of H3K9bhb within the binding pocket of ENL$_{YEATS}$. Dashed lines, hydrogen bonds; Curved brushes, hydrophobic interactions.

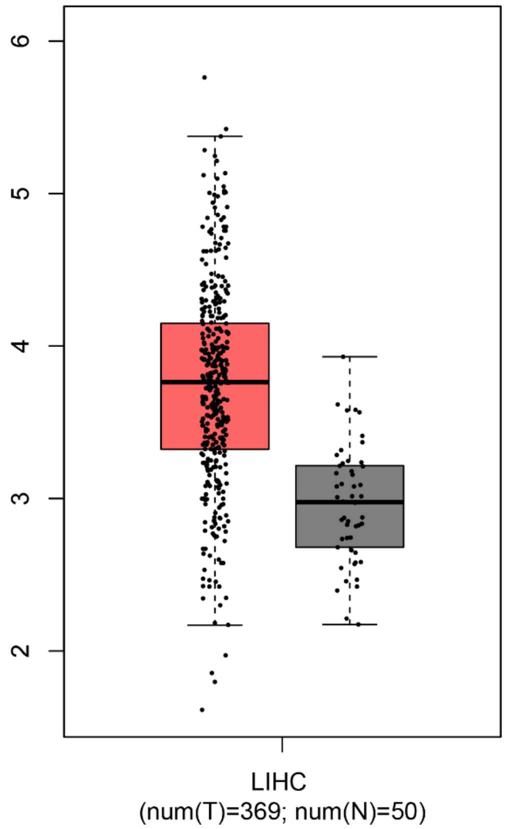

Figure S7. Box plot of liver hepatocellular carcinoma (LIHC) patients and the corresponding normal controls from the GEPIA database.

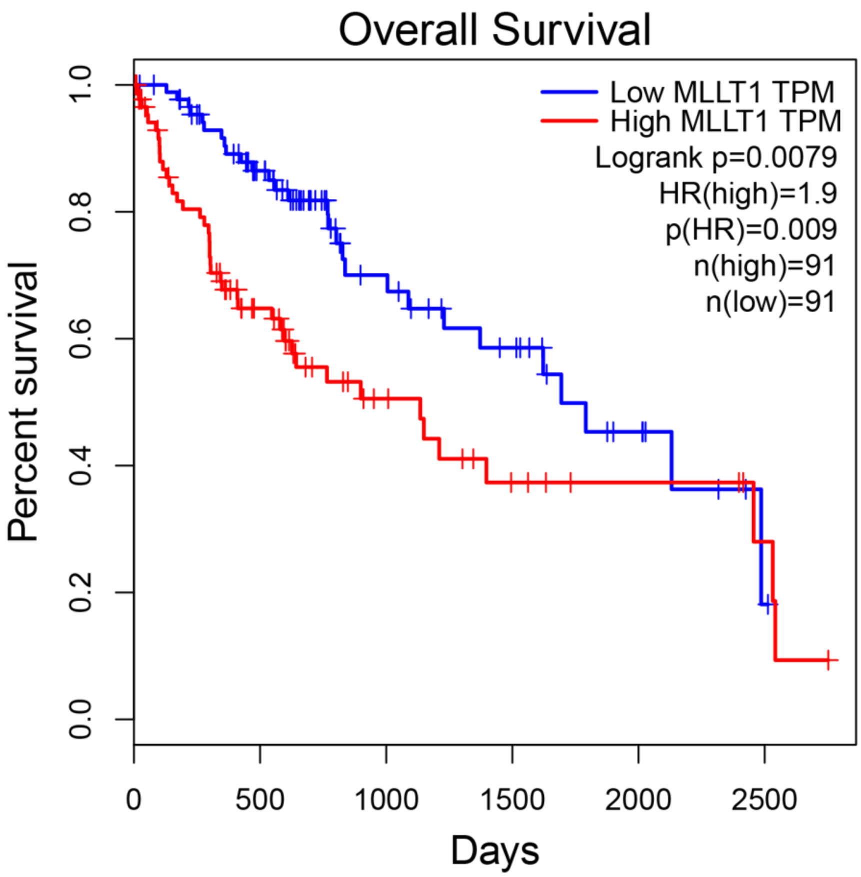

Figure S8. High expression of ENL (MLLT1) is associated with poor overall survival of patients with liver hepatocellular carcinoma (LIHC) from the GEPIA database.

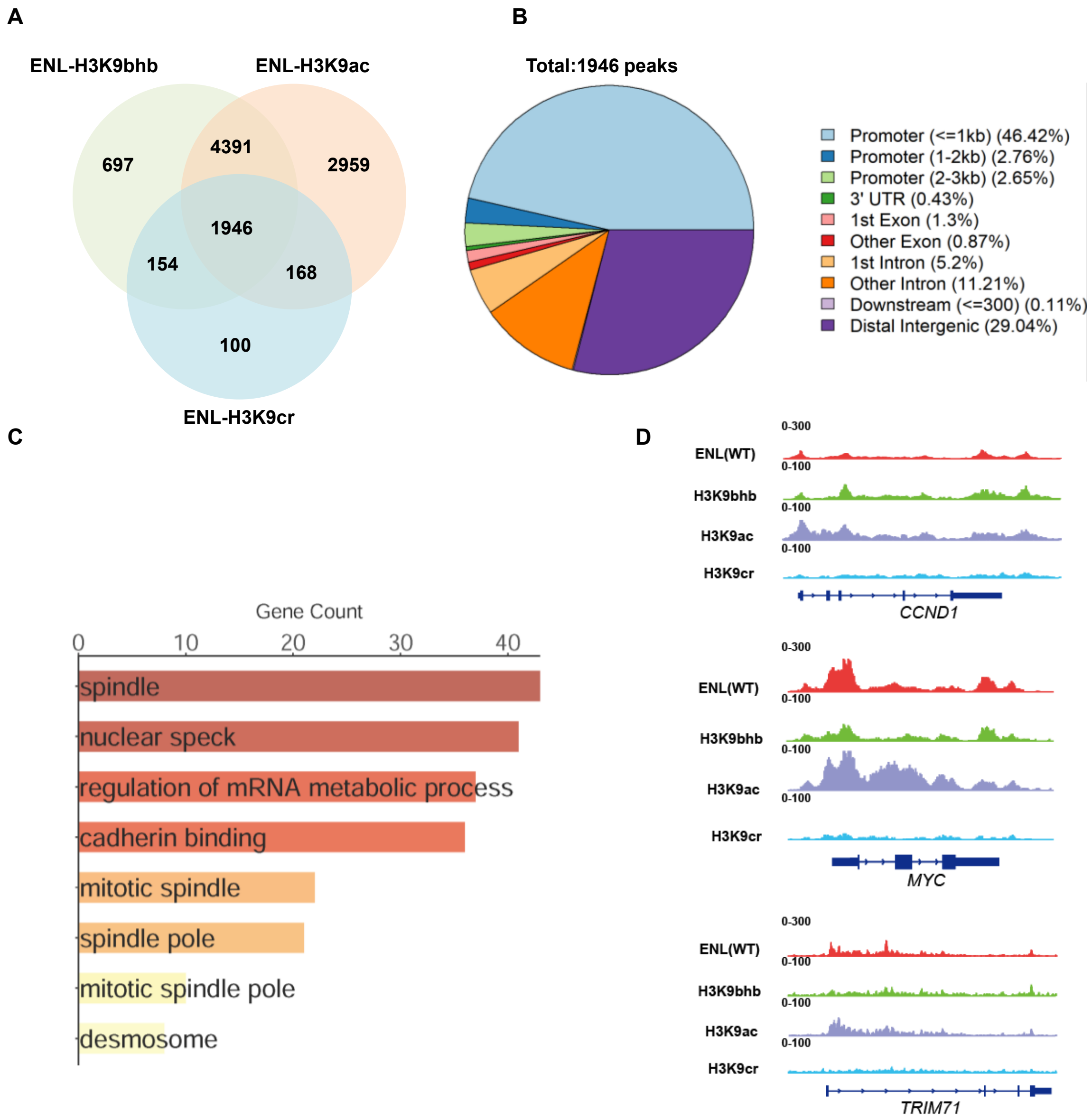

Figure S9. (A) Venn diagram showing the overlap of H3K9bhb-ENL, H3K9ac-ENL, and H3K9cr-ENL-occupied peaks in HepG2 cells. (B) Genome-wide distribution of the overlapped peaks in (A). (C) The enrichment clusters in the GO analysis of the overlapped genes (1459 genes). (D) The genome browser view of WT-ENL, H3K9bhb, H3K9ac, and H3K9cr at *CCND1*, *MYC*, and *TRIM71* locus, respectively.

Supplementary Tables

Table S1. Identified binders using probe H3K4me3 and comparative proteomic analysis.

Accession	Gene Name	Fold change	p-value	Spectral counts+0.1					
				Probe C2-1	Probe C2-2	Probe C2-3	Probe 2-1	Probe 2-2	Probe 2-3
O14646	CHD1	2.76	2.51E-05	78.1	89.1	89.1	226.1	236.1	246.1
Q9UK53	ING1	27.67	0.0013	0.1	0.1	0.1	2.1	3.1	3.1
Q96ST3	SIN3A	4.26	0.0022	2.1	1.1	1.1	7.1	6.1	5.1
Q9Y657	SPIN1	2.52	0.0075	1.1	1.1	1.1	3.1	2.1	3.1
Q9UPP1	PHF8	5.38	0.0102	2.1	3.1	2.1	9.1	13.1	17.1
Q9BTC0	DIDO1	24.85	0.0170	0.1	1.1	0.1	6.1	15.1	11.1
Q5VWG9	TAF3	64.33	0.0354	0.1	0.1	0.1	6.1	10.1	3.1
Q6P1X5	TAF2	8.74	0.0001	2.1	1.1	2.1	16.1	16.1	14.1
Q1KMD3	HNRNPUL2	2.93	0.0002	9.1	6.1	9.1	23.1	23.1	25.1
P49848	TAF6	77.67	0.0003	0.1	0.1	0.1	7.1	7.1	9.1
Q9Y2P8	RCL1	37.67	0.0004	0.1	0.1	0.1	3.1	4.1	4.1
Q8WUA2	PPIL4	3.15	0.0006	5.1	5.1	6.1	17.1	15.1	19.1
Q13724	MOGS	27.67	0.0013	0.1	0.1	0.1	3.1	2.1	3.1
Q96G21	IMP4	24.33	0.0022	0.1	0.1	0.1	2.1	2.1	3.1
O60832	DKC1	2.29	0.0023	11.1	13.1	12.1	26.1	32.1	25.1
Q14692	BMS1	3.01	0.0028	9.1	6.1	12.1	23.1	29.1	30.1
Q6UN15	FIP1L1	2.36	0.0038	7.1	10.1	7.1	16.1	21.1	20.1
Q9UPT8	ZC3H4	8.40	0.0043	2.1	4.1	1.1	19.1	16.1	26.1
Q96L58	B3GALT6	1.96	0.0048	2.1	3.1	3.1	6.1	5.1	5.1
Q8IXK0	PHC2	2.39	0.0058	6.1	5.1	6.1	14.1	11.1	16.1
Q9H4L5	OSBPL3	6.81	0.0065	0.1	1.1	3.1	11.1	11.1	7.1
Q14331	FRG1	5.35	0.0075	1.1	0.1	1.1	3.1	5.1	4.1
O75940	SMNDC1	4.85	0.0075	1.1	0.1	0.1	2.1	2.1	2.1
P98175	RBM10	2.63	0.0078	1.1	2.1	1.1	4.1	4.1	3.1
P31942	HNRNPH3	1.96	0.0078	2.1	2.1	3.1	5.1	4.1	5.1
Q99729	HNRNPAB	3.08	0.0079	3.1	1.1	1.1	5.1	6.1	5.1
Q8N9T8	KRI1	2.30	0.0093	12.1	9.1	11.1	19.1	28.1	27.1
Q13428	TCOF1	3.41	0.0120	13.1	10.1	12.1	30.1	38.1	52.1

P20226	TBP	5.55	0.0123	1.1	2.1	0.1	8.1	5.1	5.1
Q9Y224	C14orf166	5.62	0.0132	0.1	0.1	1.1	2.1	3.1	2.1
O75400	PRPF40A	2.61	0.0153	6.1	4.1	4.1	9.1	15.1	13.1
Q8NHQ9	DDX55	2.25	0.0161	9.1	10.1	7.1	20.1	15.1	24.1
O60293	ZFC3H1	9.26	0.0191	0.1	0.1	2.1	4.1	9.1	8.1
Q9NV31	IMP3	34.33	0.0194	0.1	0.1	0.1	5.1	2.1	3.1
Q7Z6E9	RBBP6	2.57	0.0241	10.1	12.1	12.1	22.1	27.1	39.1
Q14241	ELOA	3.17	0.0241	1.1	1.1	0.1	2.1	3.1	2.1
Q2NL82	TSR1	3.56	0.0254	2.1	1.1	1.1	4.1	4.1	7.1
Q12872	SFSWAP	21.00	0.0257	0.1	0.1	0.1	2.1	1.1	3.1
O43290	SART1	2.87	0.0303	2.1	4.1	6.1	8.1	15.1	12.1
Q5VTL8	PRPF38B	2.89	0.0341	1.1	2.1	2.1	4.1	7.1	4.1
Q9NX24	NHP2	3.45	0.0406	0.1	2.1	3.1	4.1	8.1	6.1
Q8WY36	BBX	2.26	0.0452	3.1	10.1	5.1	12.1	17.1	12.1
Q9UNX4	WDR3	4.08	0.0474	0.1	0.1	1.1	2.1	2.1	1.1

Table S2. Identified binders by probe H3K9bhb and comparative proteomic analysis.

Accession	Gene Name	Fold change	p-value	Spectral counts+0.1					
				Probe C1-1	Probe C1-2	Probe C1-3	Probe 1-1	Probe 1-2	Probe 1-3
Q03111	ENL	4.04	0.0249	1.1	0.1	1.1	4.1	3.1	2.1
Q9H0A0	NAT10	2.09	0.0273	50.1	107.1	103.1	208.1	196.1	140.1
Q9NVP1	DDX18	2.97	0.0290	20.1	50.1	44.1	136.1	131.1	72.1
P51114	FXR1	3.65	0.0418	16.1	16.1	16.1	76.1	70.1	30.1
Q69YN4	KIAA1429	2.26	0.0351	33.1	31.1	27.1	76.1	85.1	45.1
Q02241	KIF23	2.13	0.0175	24.1	26.1	30.1	69.1	59.1	43.1
Q9H0H5	RACGAP1	2.57	0.0265	11.1	26.1	22.1	58.1	59.1	35.1
Q14690	PDCD11	4.12	0.0294	2.1	11.1	13.1	44.1	43.1	21.1
Q12872	SFSWAP	2.10	0.0064	16.1	14.1	16.1	32.1	38.1	27.1
Q96T88	UHRF1	2.09	0.0332	13.1	13.1	16.1	33.1	35.1	20.1
Q9UBU9	NXF1	2.79	0.0297	7.1	20.1	18.1	47.1	51.1	28.1
Q86VM9	ZC3H18	3.49	0.0058	5.1	9.1	13.1	37.1	33.1	25.1
Q9H089	LSG1	5.14	0.0193	2.1	10.1	8.1	44.1	39.1	21.1
O00566	MPHOSPH10	5.69	0.0118	0.1	3.1	8.1	26.1	23.1	15.1
Q06787	FMR1	2.12	0.0421	15.1	13.1	11.1	33.1	32.1	18.1
P19447	ERCC3	5.30	0.0361	4.1	1.1	4.1	20.1	21.1	8.1
Q9H6F5	CCDC86	2.82	0.0256	2.1	15.1	8.1	26.1	26.1	19.1
Q15269	PWP2	2.07	0.0075	6.1	7.1	10.1	18.1	16.1	14.1
Q12800	TFCP2	2.15	0.0314	2.1	5.1	4.1	9.1	9.1	6.1
Q96SB4	SRPK1	3.15	0.0032	1.1	4.1	4.1	10.1	10.1	9.1
Q9NV31	IMP3	5.09	0.0020	0.1	5.1	4.1	16.1	17.1	14.1
Q7Z417	NUFIP2	2.26	0.0344	2.1	4.1	4.1	7.1	10.1	6.1
Q6NZY4	ZCCHC8	84.33	0.0354	0.1	0.1	0.1	11.1	11.1	3.1
P12268	IMPDH2	10.13	0.0206	1.1	1.1	0.1	9.1	10.1	4.1
Q9UI30	TRMT112	2.11	0.0063	8.1	14.1	10.1	25.1	23.1	20.1
Q92759	GTF2H4	5.53	0.0058	1.1	2.1	2.1	10.1	12.1	7.1
Q9Y221	NIP7	4.77	0.0474	0.1	2.1	3.1	10.1	11.1	4.1
Q96PU4	UHRF2	7.09	0.0033	2.1	0.1	0.1	5.1	6.1	5.1

Q8N567	ZCCHC9	4.02	0.0114	2.1	2.1	1.1	7.1	9.1	5.1
Q96EE3	SEH1L	2.75	0.0142	1.1	2.1	3.1	5.1	7.1	5.1
Q5C9Z4	NOM1	2.40	0.0226	3.1	3.1	3.1	8.1	9.1	5.1
Q9UKK6	NXT1	4.26	0.0061	0.1	3.1	1.1	6.1	6.1	6.1
Q8WUM0	NUP133	4.91	0.0158	0.1	1.1	1.1	3.1	5.1	3.1
Q6RFH5	WDR74	2.43	0.0213	3.1	2.1	1.1	5.1	4.1	6.1
Q9NWT1	PAK1IP1	2.40	0.0132	1.1	2.1	1.1	3.1	4.1	3.1
Q6DD87	ZNF787	21.00	0.0257	0.1	0.1	0.1	2.1	3.1	1.1
Q68DK7	MSL1	2.82	0.0257	1.1	1.1	1.1	3.1	4.1	2.1
Q99755	PIP5K1A	2.11	0.0249	1.1	3.1	2.1	4.1	4.1	5.1
O15213	WDR46	5.62	0.0132	0.1	1.1	0.1	2.1	2.1	3.1
Q6NW34	NEPRO	4.04	0.0249	0.1	2.1	0.1	3.1	3.1	3.1
Q9NVM9	INTS13	4.04	0.0249	1.1	1.1	0.1	3.1	4.1	2.1
Q9NPD3	EXOSC4	2.63	0.0352	2.1	1.1	1.1	3.1	5.1	3.1
Q9NS91	RAD18	21.00	0.0257	0.1	0.1	0.1	2.1	3.1	1.1

Table S3. The primers used for qPCR analysis.

**CCND1:**
F: AGAGCCACCTCCACCTCACC
R: TGCGGCGGAGTTGCCCCT
**MYC:**
F: TTAATTCATGCGGCTCTCTTACTC
R: TTTTCCCCCACGCCCTCT
**TRIM71:**
F: AGCCGTACGCTGCTGGAAC
R: TCGCATAATACCAAAGTGGAAAGG
